# Endometrial scratching before euploid embryo transfer: a case–control study

**DOI:** 10.1007/s10815-024-03360-x

**Published:** 2025-01-17

**Authors:** Sophia M. K. Lindekugel, Lexi Rose Frankel, Jeffrey L. Deaton, Allan Dong, Rebekah Lassiter, Ricardo F. Savaris, Bruce A. Lessey

**Affiliations:** 1 Department of Obstetrics and Gynecology, Baylor College of Medicine, Houston, TX USA; 2 Department of Obstetrics and Gynecology, Atrium Health Wake Forest Baptist, 1 Medical Center Blvd, Watlington Hall, Winston-Salem, NC 27157 USA; 3 Wake Forest University, Winston-Salem, NC 27109 USA; 4 Department of Obstetrics and Gynecology, Universidade Federal Do Rio Grande Do Sul, Porto Alegre, RS 90035-130 Brazil

**Keywords:** Endometritis, IVF-ET, Pregnancy, Endometrial scratching, Miscarriage

## Abstract

**Purpose:**

To evaluate the effect of endometrial scratching on in vitro fertilization (IVF) success rates in women undergoing frozen embryo transfer with preimplantation genetic testing for aneuploidy (PGT-A).

**Methods:**

Biopsy was performed at oocyte retrieval in cases and compared to those who did not undergo scratching (controls). Endpoints included pregnancy loss, total pregnancy, clinical pregnancy rate (CPR), ongoing pregnancy rate/live birth rate (OPR), and the incidence of chronic endometritis (CE).

**Results:**

Women in case and control groups had similar characteristics, such as age, body mass index, and parity. Overall total pregnancy rate was 82.43% following single euploid embryo transfer in cases compared to 85.7% in controls (*p* = 0.57; OR = 1.27; 95% CI = 0.565 to 2.78). CPR was 73.3% and 76.2% (*p* = 0.62; OR = 1.18; 95% CI = 0.594 to 2.29), and OPR between groups was 64.8% and 72.3% (*p* = 0.28; OR = 1.42; 95% CI = 0.74 to 2.7). First trimester loss rate was similar at 16.9%% and 15.5%%, respectively (*p* = 0.82). Subjects who underwent scratching closer to their frozen embryo transfer (≤ 3 months) had a similar overall pregnancy rate to those with a longer delay between retrieval and frozen embryo transfer (> 3 months 81.4% vs.87.5%; *p* = 0.56). CE incidence was 10.8% (8 out of 74; 95% CI = 5.5% to 19.9%). The odds of having a second trimester loss were significantly higher in the scratch group compared to controls (*p* = 0.03; OR = infinity; 95% CI = 1.3 to infinity).

**Conclusions:**

Endometrial scratching before frozen euploid embryo transfer did not influence pregnancy outcome but increased the chances of second trimester loss. CE was a frequent finding in those undergoing endometrial scratching at the time of retrieval.

## Introduction

Endometrial scratching, also commonly known as endometrial biopsy, is a procedure that intentionally injures the endometrial lining of the uterus. This procedure has been proposed as a potential intervention to improve endometrial receptivity in sub-fertile women and, in the case of in vitro fertilization (IVF), to improve the chances for successful embryo implantation and pregnancy [1, 2]. In the original study by Barash et al., endometrial scratching was performed four times within the same cycle before initiating IVF treatment [1, 2], a protocol not repeated in the subsequent studies referenced. A meta-analysis examined the efficacy of a single scratch versus two scratches within the same cycle, with findings indicating a higher pregnancy rate when two scratches were performed [3, 4].

The basis for a hypothetical effect was initially suggested by prior observations in animal models. Loeb demonstrated in 1907 that injury to the uterus induced decidualization in the guinea pig uterus [5], similar to that later observed and reported in the mouse model [6, 7]. In mice, leukemia inhibitory factor (LIF) was the first protein found to be essential for implantation [8], later also demonstrated in human endometrium [9]. Endometrial injury was shown to increase LIF expression after hysteroscopy in women [9, 10], also demonstrated in the mouse model [11]. Injury to the endometrial lining triggers an immune inflammatory response, which leads to the release of beneficial growth factors and cytokines involved in endometrial regeneration, angiogenesis, and possibly adhesion molecules involved in implantation [12, 13]. Some studies have reported increased clinical pregnancy rates (CPR) and live birth rates among women who underwent endometrial scratching, while other studies have failed to demonstrate significant benefit or report conflicting results [14–19]. Of note, a multicenter, open label, randomized, controlled trial also demonstrated no added value of endometrial scratching on improving IVF outcomes [20].

The optimal timing and technique for endometrial scratching remain controversial, with variations in the timing, method, number of scratches, and depth of injury [3, 15, 16]. In addition, the optimal length of time between biopsy and subsequent pregnancy attempt or embryo transfer has not been firmly established. As pointed out by Vitagliano et al., prior studies have been limited by the lack of genetic testing of preimplantation embryos and the failure to include chronic endometritis (CE) as a potential cause of implantation failure [14–19].

In this study, we performed a prospective case–control study of women who underwent endometrial scratching at the time of oocyte retrieval, who intended to undergo preimplantation genetic testing for aneuploid (PGT-A) of their embryos, compared to a contemporaneous control group that did not undergo endometrial scratching. Our analysis also included the assessment and treatment of CE in the scratching group, as another potential benefit of performing endometrial biopsy in women undergoing IVF with PGT-A testing and delayed frozen embryo transfer (FET).

## Materials and methods

### Study design and setting

This prospective cohort study was conducted (including recruitment, exposure, follow-up, and data collection and analysis) between January 1, 2019, and December 31, 2022, at the Center for Fertility and Reproductive Surgery at Atrium Health Wake Forest Baptist, Winston-Salem, NC, USA.

### Participants

These studies were approved by the Institutional Review Board from the Committee for the Protection of Human Subjects (IRB00079880; IRB102938).

Women aged 26 to 44 who were undergoing IVF with PGT-A testing were included. Cases comprised 74 women undergoing IVF between August 1, 2019, and November 30, 2022, who gave consent to perform endometrial scratching at the time of egg retrieval. Controls included 105 subjects who underwent IVF without scratching from January 1, 2019, to April 30, 2022, who were either retrieved outside the window of recruitment or did not consent to endometrial scratching.

The cases underwent conscious sedation, and, at the end of the IVF oocyte retrieval, a pipelle device (CooperSurgical, Inc., Trumbull, CT 06611, USA) was inserted into the endometrial cavity. Endometrium was obtained by gentle suction after the retrieval of oocytes was completed. Endometrial biopsy specimens were placed in 10% buffered formalin and transported to the Pathology laboratory of Atrium Health Wake Forest Baptist for paraffin embedding, sectioning, and examination for CE. All subjects had saline infusion sonohysterography (SIS) prior to initiating IVF.

### Stimulation for retrieval

Ovarian stimulation protocols were determined based on patient age, body mass index (BMI), anti-Müllerian hormone (AMH) levels, and previous response to gonadotropins. All patients were treated with a combination of recombinant FSH (Follistim; Merck, Rahway, NJ, USA or Gonal-f; EMD Serono, Geneva, Switzerland) and urinary FSH and LH (Menopur; Ferring Pharmaceuticals Inc., Parsippany, NJ, USA). Pituitary suppression was achieved with a gonadotropin-releasing hormone (GnRH) antagonist (Ganirelix Acetate; Merck, Kenilworth, NJ, USA; or Cetrotide Acetate; EMD Serono Inc., Geneva, Switzerland). The GnRH antagonist was initiated when the lead follicle reached a mean diameter of 13 mm or when the estradiol level was projected to be between 400 and 1100 pg/mL. This suppression continued until the administration of the trigger shot for final oocyte maturation. Patients were monitored every 2–3 days with transvaginal ultrasound and serum estradiol and progesterone levels.

The final trigger shot for oocyte maturation was determined based on the risk of ovarian hyperstimulation syndrome (OHSS). Patients with a high AMH level (> 4.5 ng/mL), a concomitant high estradiol level (> 3000 pg/mL), and/or a large number of secondary follicles (10–14 mm in diameter), along with a normal or elevated baseline luteinizing hormone (LH) level, were given a GnRH agonist trigger (40 units of Lupron; AbbVie Inc., Chicago, IL, USA). Patients with an AMH level < 3.0 ng/mL were typically given an hCG trigger (10,000 units of Pregnyl; EMD Serono, Geneva, Switzerland; or Novarel; Ferring Pharmaceuticals Inc., Parsippany, NJ, USA). Based on AMH levels and final estradiol concentrations, some patients received a co-trigger consisting of both hCG and a GnRH agonist. The hCG dose ranged from 1500 to 5000 IU depending on clinician concerns regarding OHSS.

Transvaginal oocyte retrieval was performed under conscious sedation 36 h after the final trigger for oocyte maturation. The retrieved oocytes were incubated for 2–3 h before deciding whether to perform intracytoplasmic sperm injection (ICSI) or allow conventional fertilization (i.e., placing approximately 50,000 sperm in a small droplet of media under oil with each oocyte). This decision was based on sperm quality, the number of retrieved eggs, and the couple’s diagnosis.

For ICSI procedures, cumulus/corona cells were removed using hyaluronidase and manual stripping. Each metaphase II oocyte was injected with a single spermatozoon. Fertilization was assessed under an inverted microscope 18–22 h after ICSI or conventional fertilization by identifying one or more pronuclei and polar bodies. Oocytes with two distinct polar bodies and two clear pronuclei (2PN) were considered normally fertilized.

Normally fertilized oocytes were cultured uninterrupted from days 1–6 in complete embryo culture media (Cooper Surgical; Trumbull, CT, USA). Resultant good quality blastocysts underwent trophectoderm biopsy under standard conditions. Blastocysts were graded according to the quality of the inner cell mass (ICM) cells, trophectoderm cells, and the degree of expansion per the Gardner classification system. Good-quality blastocysts were defined as having a blastocele volume > 80% of the embryo with healthy ICM cells (A-B) and healthy trophectoderm cells (A-B). Trophectoderm biopsy samples were sent to a next-generation sequencing laboratory (IGenomix; Miami, FL, USA) for genetic analysis and reported as euploid, aneuploid, high- or low-level mosaicism, no result, or chaotic.

### Frozen embryo transfer (FET) procedures

Endometrial preparation for FET was performed using one of two methods. Most patients underwent endometrial stimulation with a combination of oral Estrace® (AbbVie Inc., North Chicago, IL, USA) and Vivelle-Dot® patches (Novartis Pharmaceuticals Corporation, East Hanover, NJ, USA), using a 0.1 mg patch every 3–4 days. Patients unable to tolerate either the patch or oral estrogen were treated with oral Estrace® alone at a dose of 1 mg twice daily. During stimulation, approximately 2–3 ultrasounds were performed: one at baseline and one or two during mid-cycle. Once a qualified endometrium was reached (at least 7 mm in thickness with a trilaminar appearance), progesterone in oil (50 mg intramuscularly) was started daily in the morning. The blastocyst was thawed on the morning of the sixth day of progesterone administration. Patients who did not achieve a qualifying endometrial lining on this protocol were offered a modified natural cycle as described below. Oligo-ovulatory patients were offered either an aromatase inhibitor protocol or gonadotropin stimulation based on provider preference.

Some patients chose a modified natural cycle protocol. In the modified natural cycle protocol, mid-cycle transvaginal ultrasound was used along with estradiol, progesterone, and luteinizing hormone (LH) levels to identify a dominant follicle. When a follicle of at least 17 mm was observed, along with an estradiol level > 150 pg/mL, progesterone level < 2.0 ng/mL, LH level < 15 IU/L, and an endometrium ≥ 7 mm in thickness with a trilaminar appearance, a trigger shot of hCG (10,000 IU) was administered in the evening. The blastocyst was thawed on the morning of the seventh day following the trigger shot. Luteal support was provided with daily progesterone in oil (50 mg IM), starting 2 days after the trigger shot. Cycles with elevated mid-cycle progesterone levels (> 2 ng/mL) were canceled. Cycles with an LH level > 15 IU/L were adjusted so that embryo transfer occurred on the sixth day of progesterone administration. A GnRH antagonist was used in modified natural cycles at the provider’s discretion.

Approximately 60% of FET cycles were programmed estrogen/progesterone cycles, with similar proportions in both groups (scratching and controls). Blastocysts were vitrified using a multistep Irvine Scientific Vitrification Freeze Kit at room temperature and thawed using an Irvine Scientific Vitrification Thaw Kit (Irvine Scientific Inc., Santa Ana, CA, USA). Thawing involved placing the blastocysts in a thawing solution at 37 °C for 60 s, followed by transfer to equilibrated 20% Irvine Continuous Single Culture-NX Complete media (Irvine Scientific Inc., Santa Ana, CA, USA).

Single embryo transfer was performed in the afternoon of the blastocyst thawing at approximately 126 h of progesterone administration. The procedure was conducted under ultrasound guidance with a full bladder using a Wallace Sure View Soft catheter (CooperSurgical Inc., Trumbull, CT, USA). The embryo was transferred 1.5 cm from the fundus using a standard three-handed technique. Prior to transfer, cervical mucus was flushed out and a mock catheter was passed to ensure proper placement. After transfer, the catheter was checked; if it was not clear, a second transfer attempt was performed. Patients remained recumbent for 15 min before being discharged to resume normal activities.

Serum hCG and progesterone levels were measured 8–10 days after embryo transfer. If hCG results were positive, repeat testing occurred 2–4 days later, followed by transvaginal ultrasound at 7 weeks’ gestation. If fetal heartbeat and appropriate crown-rump length were observed, patients were referred for obstetrical care.

### Treatment for chronic endometritis

First-line treatment for CE was oral doxycycline at a dosage of 100 mg twice daily for 10 days. In resistant cases requiring repeat treatment, we used oral ciprofloxacin (400 mg) and metronidazole (500 mg), both administered twice daily for 14 days. Ideally, patients with this diagnosis should undergo a test of cure, after which embryos can be transferred in the next available cycle.

### Variables

Groups were divided into control and cases (scratched), and the variables were (1) total pregnancy, considering all cases that resulted in pregnancy (biochemical pregnancy, “blighted ovum,” ectopic pregnancy, miscarriage in the first trimester, and miscarriage in the second trimester); (2) clinical pregnancy rate (CPR) defined as when a gestational sac or embryo/fetal heart rate was observed in the uterus; (3) ongoing pregnancy/live birth rate (OPR), defined as a pregnancy of more than 22 weeks or delivered of a fetus; (4) first trimester loss; (5) second trimester loss; (6) euploid embryos, as described in The International Glossary on Infertility and Fertility Care, 2017 [21]; (7) age; (8) BMI; (9) time between biopsy and FET; (10) parity; and (11) presence of CE. CE was defined as the presence of two or more plasma cells in 10 high-power fields [22].

### Data sources/measurements

Data were obtained from the SART database and institutional medical records. Most variables were measured as percentages, except for age and BMI, which were measured in years and kg/m^2^, respectively. The time between biopsy and frozen embryo transfer was measured in days. First and second trimester miscarriages were analyzed as a percentage of all pregnancies in each group. CE, when diagnosed by pathology, was defined as present or absent. 

### Bias

Patient selection was temporally related but not randomized, introducing potential unintentional selection bias. Pathologists were blinded to the study when evaluating samples from the endometrial scratching group. Pregnancy tests and outcomes were assessed independently of knowledge about patient’s group.

### Study sample size

Sample size was calculated according to the literature [23, 24]. At least 60 patients per group were required to achieve 90% power with a 5% alpha-error to detect an increase in live birth rate from 27% in the control group to 55% in the endometrial scratch group.

### Quantitative variables

Age and BMI were measured as mean and standard deviation, while the remaining variables were measured as percentage.

### Outcome measures

Outcomes were measured as individual numbers and included no pregnancy, total pregnancy rate, CPR, OPR, first trimester loss, second trimester loss, time between biopsy and frozen embryo transfer (in days), and presence of CE (yes/no). Demographic and cycle characteristics were also compared between cases and controls, including age (years), BMI (kg/m^2^), parity (number of times a woman has given birth to a fetus with a gestational age of 20 weeks or more, regardless of whether the infant was born alive or was stillborn), and nulliparous (a woman who has never completed a pregnancy beyond 20 weeks’ gestation). The effect of cycle preparation type on outcomes was not included in our analysis.

### Statistical analysis

Age and BMI were treated as continuous variables. Outcomes of CPR (pregnant, non-pregnant), live birth rate, number of euploid embryos, and first and second trimester loss rates were compared between cases and controls using non-parametric tests. The time between biopsy and frozen embryo transfer varied among subjects and was categorized as a binary variable: short duration (< 90 days) or long duration (≥ 90 days) between retrieval and transfer. These two groups were then compared regarding outcomes.

Chi-square for trend and chi-square test were used for comparisons of categorical data. Parametric data were compared between groups using Student’s *t*-test if data had a Gaussian distribution. Gaussian distribution was verified by the D’Agostino and Pearson omnibus normality test. Mann–Whitney U test was used if a Gaussian distribution was not present. Odds ratios (ORs) with 95% confidence intervals (CIs) were calculated.

Statistical analysis was performed with GraphPad Prism version 10.3 for Mac (GraphPad Software, La Jolla, CA, USA).

## Results

### Participants and descriptive data

All the included subjects completed a single euploid embryo for transfer. In total, 74 subjects consented for endometrial scratching (cases), and 105 were included as controls. Demographic variables are presented in Table 1.

**Table 1:** Demographic data from cases (scratching) and controls

Demographics	Case (*n* = 74)	Control (*n* = 105)	*p*
Age (mean, SD)	35.51 (3.7)	35.25 (3.8)	0.64^a^
BMI (mean, SD)	28.29 (6.4)	26.46 (5.7)	0.076^d^
Parity			
Nulliparity/parous (*n*)	54/20	89/16	0.52^c^
Race			
White	55	75	0.74^b^
Black	5	9	
Asian/other	14	21	
Euploid embryos (mean; SD)	5.18 (3.8)	4.8 (3.5)	0.55^d^
Oocytes retrieved (mean, SD)	18.5 (9.8)	19.7 (9.2)	0.22^d^

*BMI*, body mass index; *SD*, standard deviation

^a^Student’s *t*-test

^b^Chi-square for trend test

^c^Chi-square test

^d^Mann-Whitney test

### Outcome data

Pregnancy outcomes for cases and controls following frozen embryo transfer were not significantly different (Table 2), except for the second trimester loss rate. The odds of having a second trimester loss were significantly higher in the scratch group (3 out of 61; 4.9%; 95% CI = 1.6 to 13.4%) compared to controls (0 out of 90; 0%; 95% CI = 0 to 5.1%; *p* = 0.03; OR = infinity; 95% CI = 1.3 to infinity).

**Table 2:** Pregnancy outcomes for cases (scratching) and controls following frozen embryo transfer

Category	Case (*n* = 74)	Control (*n* = 105)	*p*	Odds ratio (95% CI)
Total pregnancy	61 (82.43)	90 (85.70)	0.55^a^	1.27 (0.56 to 2.78)
Biochemical	6	7	0.7^a^	1.23 (0.39 to 3.49)
Short/long^d^	4/2	6/1		
“Blighted ovum”	1	2	0.7^a^	0.7 (0.048 to 6.16)
Short/long^d^	1/0	2/0		
Ectopic pregnancy	0	1	0.3^a^	0 (0.0 to 12.77)
Short/long^d^	0/0	1/0		
First trimester loss (miscarriage) *n* (%)^b^	3	4	0.9^a^	1.06 (0.26 to 4.07)
Short/long^d^	2/1	4/0		
Second trimester loss *n* (%)^c^	3	0	0.03^a,b^	∞ (1.24 to infinity)
Short/long^d^	3/0	0/0		
Ongoing pregnancy/live birth rate *n* (%)^e^	48	76	0.2^a^	0.68 (0.37 to 1.35)
Clinical pregnancy rate ^f^	54 (73.3)	80 (76.2)	0.62^a^	0.84 (0.44 to 1.68)
Ongoing pregnancy/live birth rate ^f^	48 (64.8)	76 (72.3)	0.28^a^	1.7 (0.37 to 1.35)
First trimester loss rate ^f^	10 (13.5)	14 (13.3)	0.97^b,c^	1.01 (0.42 to 2.45)
Short/long	7/3	13/1		

^a^Chi-square test

^b^First trimester loss included biochemical pregnancy, “blighted ovum,” ectopic pregnancy, and first trimester miscarriage, and it was calculated considering all cases that got pregnant (case = 61, control = 90)

^c^Second trimester loss was calculated considering all cases that got pregnant (case = 61, control = 90)

^d^Short/long refers to the time frame between frozen embryo and its transfer into the uterus. Short (< 90 days), long (≥ 90 days)

^e^Ongoing pregnancy/live birth rate was calculated considering all cases that got pregnant (case = 61, control = 90)

^f^Calculation considered total numbers by group (case = 74 | control = 105) as denominator

Numbers are *n* (%), unless otherwise stated

As the timing between scratching and transfer might impact outcomes, we compared those with a transfer within 90 days of retrieval to those with delayed transfer (i.e., greater than 90 days). The total pregnancy, CPR, and OPR rates were similar between short and long transfer time periods (Table 3).

**Table 3:** Pregnancy outcomes for cases (scratching) and controls following frozen embryo transfer considering short (< 90 days) or long (≥ 90 days) time of transferring

Category	Case	Control	*p*^a^
Short	(*n* = 59) Preg/not preg	(*n* = 84) Preg/not preg	
Total pregnancy rate	48/11	75/9	0.17
Clinical pregnancy rate	43/16	66/18	0.43
Ongoing pregnancy/live birth rate	38/21	62/22	0.22
Long	(*n* = 15) Preg/not preg	(*n* = 21) Preg/not preg	
Total pregnancy rate	13/2	15/6	0.27
Clinical pregnancy rate	11/4	14/7	0.66
Ongoing pregnancy/live birth rate	10/5	14/7	1

^a^Chi-square test

Numbers are *n* unless otherwise stated. *Preg* pregnant, *not preg* not pregnant

### Chronic endometritis

Eight of 74 (10.8%; 95% CI = 5.5 to 19.9%) biopsy samples returned with a diagnosis of CE. Of these, one subject was not treated, and one subject did not undergo a test of cure after antibiotic treatment. The overall OPR in the endometritis group was 4 of 8 (50%; 95% CI = 21.5 to 78.4%). First trimester loss rate was 3 of 8 (37.5%; 95% CI = 13.6 to 74.1%). Second trimester loss was observed in 1 of 8 (12.5%; 95% CI = 2.2 to 53.3%). Neither of the subjects who were not treated or did not have a test of cure conceived after FET with euploid embryos in our study.

## Discussion

Endometrial biopsy at the time of oocyte retrieval provides a convenient opportunity for scratching before subsequent PGT-A testing and planned euploid embryo transfer. Patients are typically sedated minimizing the discomfort usually associated with endometrial biopsy. Additionally, endometrial sampling during retrieval allows for evaluation of the endometrium to rule out endometritis. In this study, we report that endometrial scratching at the time of oocyte retrieval did not impact subsequent IVF outcomes when transferring PGT-A-tested euploid embryos. Similar to other studies that found no effect, our study did not adhere to the original protocol of performing four separate scratching procedures prior to initiating IVF.

### Pregnancy outcomes

We found that OPR was present in 48 out of 74 patients in the scratch group (64.8%; 95% CI = 53.5 to 74.7%), while in the control group, it was present in 76 out of 105 patients (72.3%; 95%CI = 63.1 to 80%), evidencing no significant difference between groups. These results are in accordance with those published previously [25, 26] and represent the first study on endometrial scratching prior to euploid embryo transfer.

Our findings contrast with those published in 2003 by Barash et al. These authors reported that local injury to the endometrium doubled success rates in patients undergoing IVF [1]. They found improvements in biochemical pregnancy rates, clinical pregnancy rates, and live birth rates among women with prior implantation failure following multiple endometrial biopsies during a spontaneous cycle 1 month before their planned IVF cycle [1]. Papanikolaou et al. reported that endometrial scratching during hysteroscopy, before embryo transfer, improved pregnancy and live birth rates [27]. El-Toukhy et al. noted significant improvements in clinical pregnancy rates (CPR) and OPR after local injury in non-randomized studies [28]. In a more recent analysis, Iakovidou et al. reported a benefit for OPR from endometrial scratching but not for ongoing pregnancy compared to placebo/sham or no intervention [29]. Potdar et al. published a systematic review and meta-analysis on endometrial injury for recurrent implantation failure, noting a 70% higher clinical pregnancy [30].

Possible explanations for these discrepancies could be related to methodological issues. For instance, Barash et al. analyzed a population consisted in women who failed to conceive during one or more cycles of IVF and embryo transfer (ET), performing multiple biopsies on days 8, 12, 21, and 26 of the spontaneous menstrual cycle before IVF-ET treatment [1]. In Papanikolaou’s meta-analysis, endometrial biopsies were performed via hysteroscopy at various times (4 to 14 days) prior to the subsequent ART cycle [27]. The differing results from Potdar et al.’s study could stem from their population of women with recurrent implantation failure in non-IVF cycles [30], which differs from our population.

In this study, we included only women who underwent transfer of a single euploid embryo after an IVF-PGT-A testing. The lack of benefit we report may reflect differences in our study population compared to prior studies. A systematic review by Vitagliano et al. indicated that endometrial scratch injury was beneficial in women with two or more embryo transfer failures [3]. A subsequent meta-analysis from 2023 reported greater benefits from scratching with an increasing number of prior failures (RR = 1.35; 95% CI = 1.20 to 1.53) [29]. Our population did not include prior IVF failure as part of our selection criteria.

### Endometritis

CE incidence was 10.8% among patients who had an endometrial biopsy at the time of oocyte retrieval. CE is a chronic infectious disease of the endometrium that is more prevalent in women with infertility and recurrent implantation failure [31, 32]. Vitagliano et al., in a systematic review, found a significantly higher ongoing live birth rate (OR 6.81; 95% CI = 2.08 to 22.24), CPR (OR 4.98; 95% CI = 1.72 to 14.43), and implantation rate (OR 3.24; 95% CI = 1.33 to 7.88) in patients with treated and cured CE compared to those with persistent CE. While severe endometritis reduces live birth rates in IVF, mild cases do not significantly alter outcomes [33]. In our study, we report that 8 of 74 (10.8%) had signs of endometritis on their endometrial biopsy; however, severity was not assessed in our results. Of the eight cases detected, one untreated subject did not conceive, and another who did not have a “test of cure” also failed to conceive.

Based on Vitagliano et al.’s systematic review, detection and treatment of CE remain understudied and should be considered essential areas for quality improvement, especially regarding recurrent implantation failure [33]. The relatively high prevalence of CE found in our biopsies supports the argument for routine endometrial sampling prior to frozen embryo transfer (FET), particularly after previous implantation failures with euploid embryo transfers. Ideally, CE should be ruled out before initiating IVF in any patient with unexplained infertility.

Unlike testing for endometrial receptivity, endometrial sampling to rule out CE can be performed during either the proliferative or secretory phase of the menstrual cycle. The lack of information about CE in many prior studies, including the recent randomized controlled trial by Lensen et al. [20], represents missed opportunities to enhance our understanding of this issue.

Strengths of this study include its prospective study design and inclusion of only PGT-A testing single embryo transfers. To our knowledge, this is the first study to compare scratching effects in cycles with PGT-A-defined euploid embryos. Most prior studies are limited to IVF before PGT-A testing, and only a few have included endometritis as a diagnostic finding in the setting of endometrial scratching [3]. Additionally, our sample size was relatively large when comparing scratching outcomes to controls.

Limitations include the lack of randomized selection of patients. We report negative results rather than improvement, based on our original hypothesis as stated in the sample size calculation. We did not conduct a cost–benefit analysis for adding an endometrial biopsy procedure.

The subjects included were not selected based on recurrent implantation failure; hence, they may not represent the ideal population that would optimally benefit from endometrial scratching at oocyte retrieval. Finally, like most studies evaluating endometrial scratching, this research did not conform to the original protocol involving four scratches within a single cycle before initiating IVF treatment, leaving this methodological deviation open for debate.

Future studies will likely need to focus on euploid embryo transfer failure subjects as the gold standard for endometrial receptivity investigations. The effect of CE severity and treatment will be another focus for IVF outcomes research going forward.

## Data Availability

All data supporting these findings are available by request to the author(s) or at the following link: https://docs.google.com/spreadsheets/d/1pfcfGoZnQ8GtLzcS5PbBve6IHIf51WWO1XLCOO-fj8s/edit?usp=sharing. All identifying information has been omitted to protect patient confidentiality.
